# Percutaneous embolization of lymphatic leakage after pelvic lymphadenectomy

**DOI:** 10.31744/einstein_journal/2025RC1593

**Published:** 2025-10-03

**Authors:** Raphael Freitas Rafael, Mariana Berquo Peleja, Guilherme Marcelino de Miranda, Rodrigo Gobbo Garcia, Eduardo Vieira Motta, Guilherme Moratti Gilberto

**Affiliations:** 1 Hospital Israelita Albert Einstein São Paulo SP Brazil Hospital Israelita Albert Einstein, São Paulo, SP, Brazil.

**Keywords:** Lymph node excision, Lymphatic diseases, Embolization, Morbidity

## Abstract

Lymphatic leakage after pelvic lymphadenectomy is a rare complication. Furthermore, in some cases, drainage is not possible or proves insufficient to resolve this complication. Therefore, more effective and minimally invasive interventional treatments are necessary. Accordingly, this case report presents a case of transafferent nodal embolization in a 50-year-old woman who underwent videolaparoscopic total hysterectomy with pelvic lymphadenectomy. The patient developed high post-surgery vaginal flow due to a lymphatic fistula, which was identified after intranodal lymphangiography and treated successfully via transafferent nodal embolization. This case report shows the importance of considering lymphatic leakage as a possible pelvic surgery complication, particularly when accompanied by local lymphadenectomy. Moreover, the case report reveals that intranodal lymphangiography and transafferent nodal embolization are safe and highly effective methods to detect and treat this complication. This prevents the need for follow-up surgery, which may add morbidity to an otherwise uneventful postoperative period.

**Level of evidence:**

Level 5, Case Report.

## INTRODUCTION

Lymphatic leakage after pelvic lymphadenectomy is a rare complication that occurs in up to 1.8% of patients undergoing this procedure for cervical cancer treatment.^(1)^ A high percentage of these patients can be conservatively treated.^(2)^ In other cohorts, percutaneous drainage, with or without the use of sclerosing agents, was necessary, with a high success rate of at least 80%.^(2,3)^ In cases where drainage is not possible or when it proves insufficient to resolve this complication, minimally invasive techniques, such as intranodal lymphangiography (INL) with transafferent nodal embolization (TNE) may easily decrease leakage or drain output and eventually allow for removal of the catheter within a median span of six days.^(4)^ Herein, we present the case of INL and TNE treatment in a 50-year-old woman after pelvic lymphadenectomy.

## CASE REPORT

**Figure 1:**
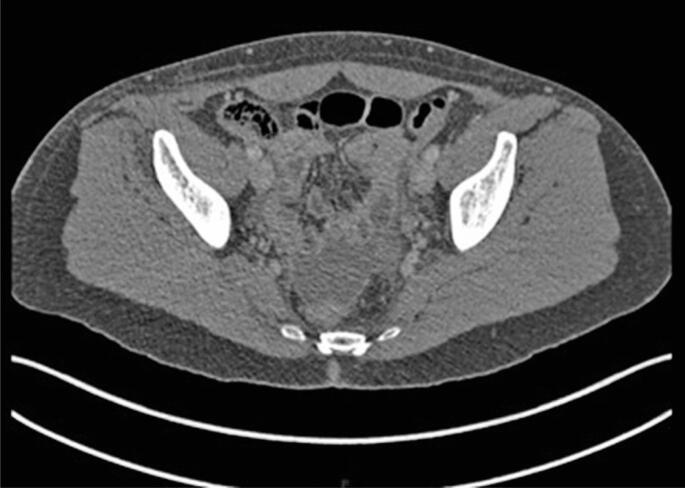
Abdominal computed tomography image showing free fluid in the pelvis. Image was generated during the investigation of possible urinary leakage

A 50-year-old woman underwent videolaparoscopic total hysterectomy with pelvic lymphadenectomy for grade I endometrioid carcinoma. She was discharged two days after the procedure and developed continuous translucent vaginal flow the following day, with a high output of over 500mL/day. Contrast-enhanced computed tomography revealed a small amount of free pelvic fluid (Figure 1), and the gynecology team initially suspected an iatrogenic ureteral lesion. On the seventh postoperative day, the urology consultant placed bilateral double-J catheters that did not alter the output of the vaginal discharge. This effectively eliminated the suspicion of a ureteral lesion.

**Figure 2:**
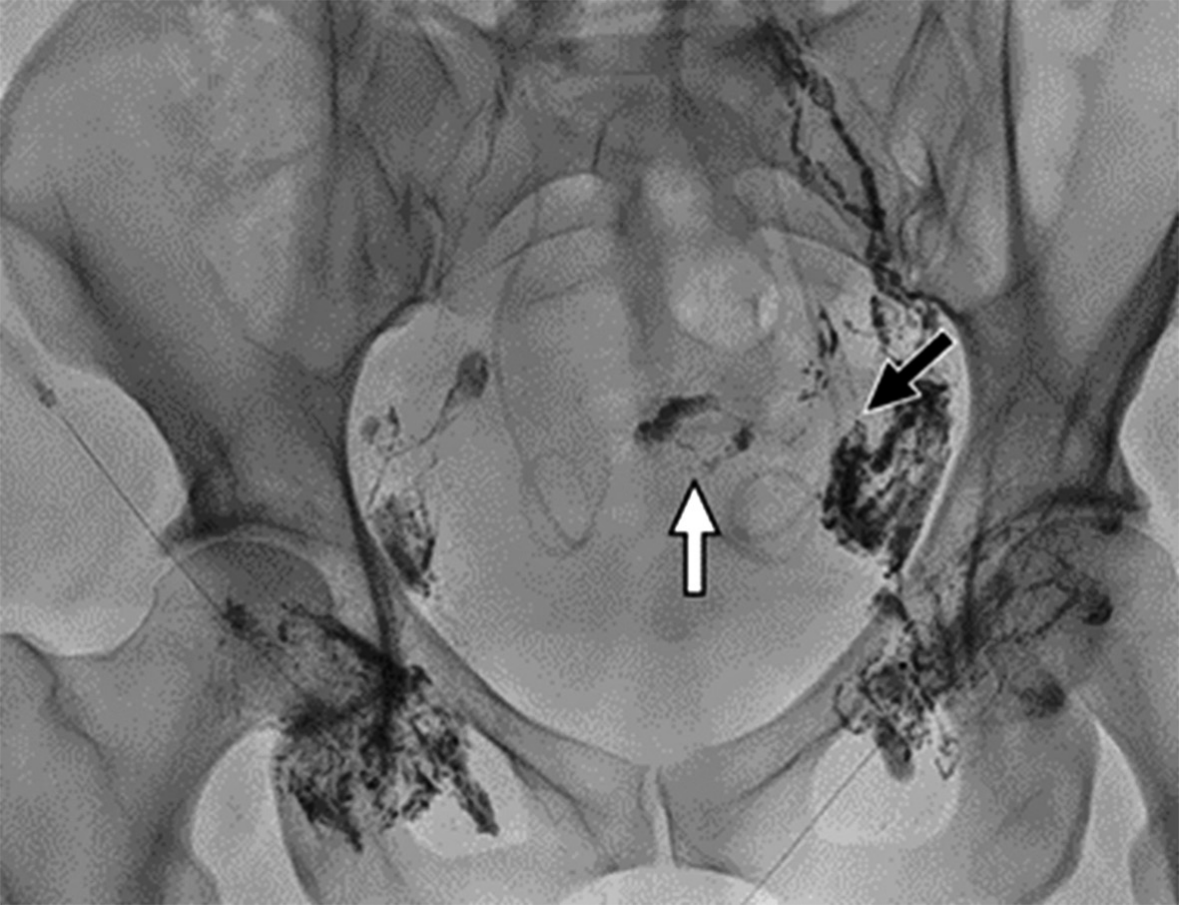
Intranodal lymphangiography shows dilated lymphatic ducts and leakage to the pelvic cavity through a small lesion to a minor pelvic lymphatic channel on the left side (black arrow). A small amount of ethiodized oil is collected in the pelvic cul-de-sac (white arrow). In contrast, lymphatic ducts on the right side show normal size and no sign of extravasation of the contrast media

**Figure 3:**
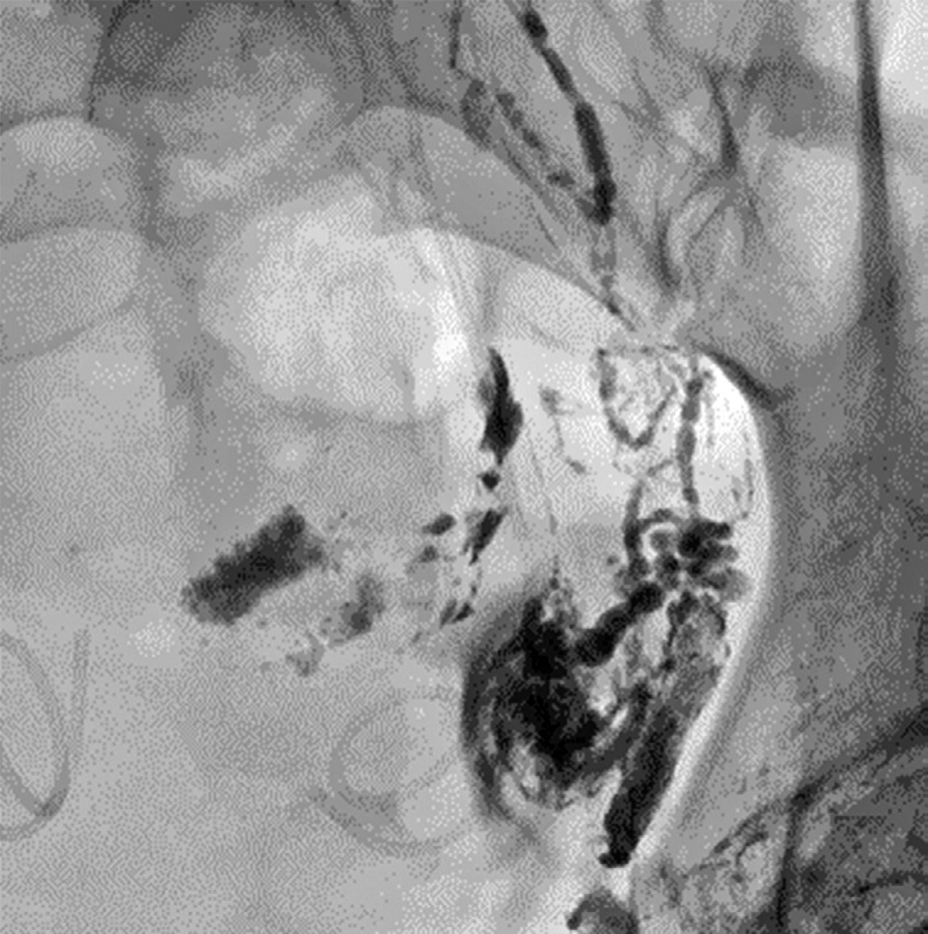
Detailed image of the left iliac fossa shows elarged lymphatic ducts and leakage to the pelvic cavity through a small lesion to a minor pelvic lymphatic channel

Seven days later, the interventional radiology team was asked to determine possible diagnostic and therapeutic options. The team ultimately chose to perform INL via bilateral ultrasound-guided puncture of the inguinal lymph nodes. INL revealed dilated lymphatic ducts and leakage into the pelvic cavity through a small lesion into a minor pelvic lymphatic channel on the left side (Figures 2 and 3).

**Figure 4:**
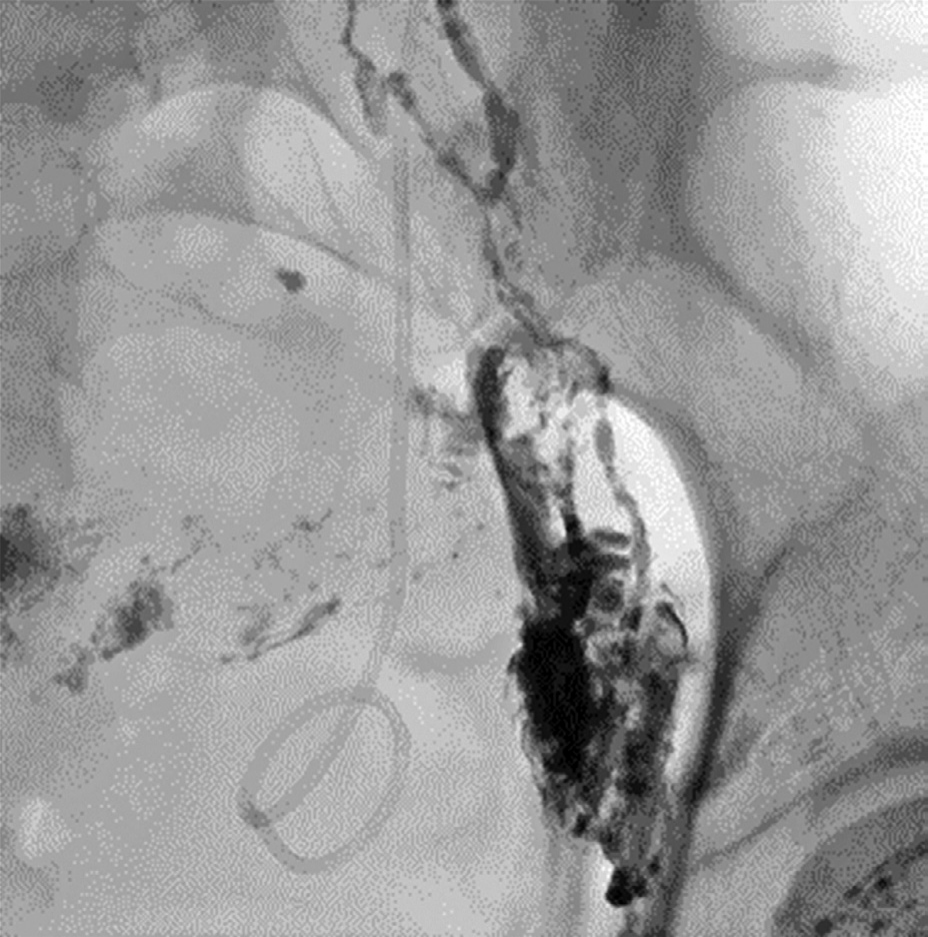
Control image after embolization with N-acetyl cyanoacrylate shows retention of the embolized agent inside lymphatic channels, with no further extravasation into the pelvic cavity

The right side showed adequate progression of the contrast medium (ethiodized oil) to the cisterna chyli. This side was then chosen to gain access to a closer lymph node via direct fluoroscopy-guided puncture with a 25G Chiba needle. A solution of N-acetyl cyanoacrylate and ethiodized oil (1:1) was then slowly injected (Figure 4). A few minutes later, the control INL showed no further extravasation of contrast media.

**Figure 5:**
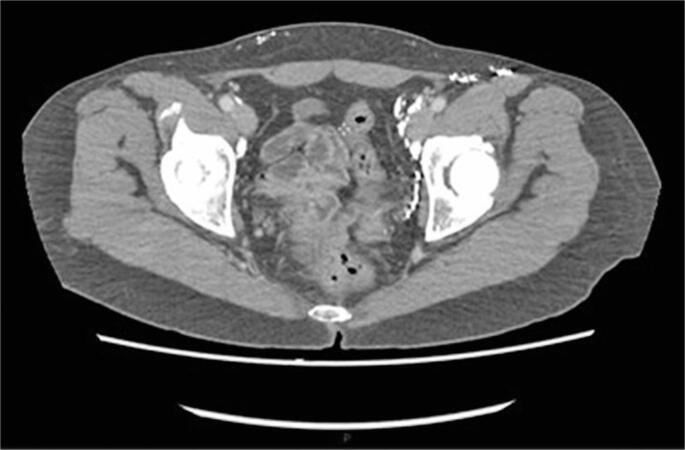
Axial view of control computed tomography image generated approximately six months after transafferent nodal embolization (TNE). The image shows the absence of free fluid in the pelvis and the TNE-related radiopaque material next to the pelvic and inguinal retroperitoneal lymph nodes

**Figure 6:**
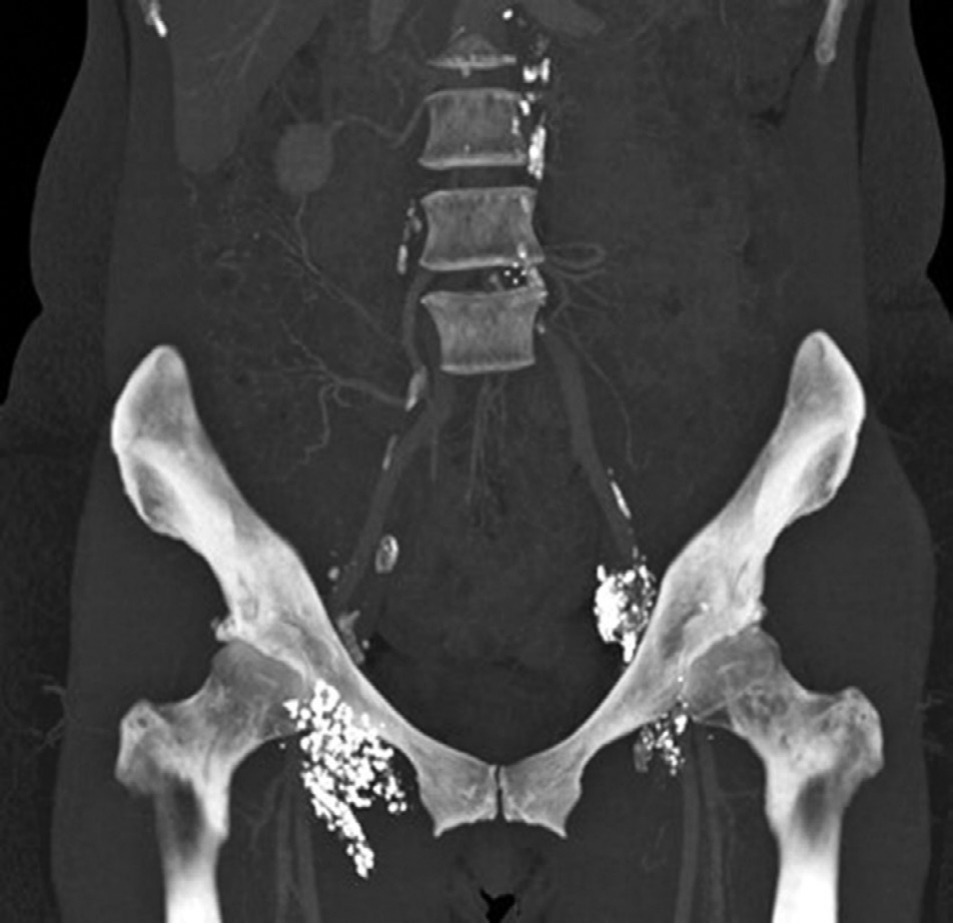
Coronal view of control computed tomography image generated approximately six months after transafferent nodal embolization (TNE). The image shows TNE-related radiopaque material next to the inguinal lymph nodes

Vaginal discharge decreased throughout the day and resolved the day after the procedure. This proved that the discharge was due to a fistula and lymphatic leakage via the unhealed vaginal vault. Figures 5 and 6 show the absence of free fluid in the pelvis and the TNE-related radiopaque material adjacent to the pelvic and inguinal retroperitoneal lymph nodes.

Informed consent was obtained from the study participant, and the study was conducted in accordance with the principles of the Declaration of Helsinki. Approval was granted by the Ethics Committee of *Hospital Israelita Albert Einstein* (CAAE: 81263624.1.0000.0071; # 6.960.995).

## DISCUSSION

Lymphorrhea is defined as free lymphatic fluid in the peritoneal cavity. This complication occurs in 0.7% and 3.1% of patients who undergo laparoscopic and open surgery, respectively, for systematic pelvic lymphadenectomy during endometrial cancer staging.^(3)^ Although lymphorrhea is rare, it may pose critical diagnostic and therapeutic challenges, as seen in this case. When lymphatic leakage does not accumulate in a lymphocele, the lymphatic leakage may not be amenable to percutaneous drainage, which is often the first diagnosis or treatment choice when conservative measures are unsuccessful. In such settings, INL and TNE are innocuous and highly effective diagnostic and treatment modalities. Moreover, in the absence of N-acetyl cyanoacrylate (ethiodized oil-lymphangiography alone), clinical success was still achieved in 88% of patients, with a mean of 1.4 interventions.^(5)^

## CONCLUSION

This case report highlights the importance of considering lymphatic leakage as a possible pelvic surgery complication, particularly when accompanied by local lymphadenectomy. Furthermore, the case report demonstrates that INL and TNE are safe and highly effective methods to detect and treat this complication. This prevents the need for follow-up surgery, which may add morbidity to an otherwise uneventful postoperative period.
